# How Does Social Capital Affect Residents’ Waste-Separation Behavior? Evidence from China

**DOI:** 10.3390/ijerph19063469

**Published:** 2022-03-15

**Authors:** Yexin Zhou, Hongke Song, Xiaopei Huang, Hao Chen, Wei Wei

**Affiliations:** 1 Center for Innovation and Development Studies, Beijing Normal University, Zhuhai 519087, China; zhouyexin@bnu.edu.cn; 2 School of Economics and Resource Management, Beijing Normal University, Beijing 100875, China; huangxiaopei@mail.bnu.edu.cn (X.H.); hchen@bnu.edu.cn (H.C.); 3 Beijing Key Lab of Study on Sci-Tech Strategy for Urban Green Development, Beijing Normal University, Beijing 100875, China; 4 School of Economics and Management, Inner Mongolia University, Hohhot 010021, China; songhongke@126.com; 5 Management Academy of China Cooperatives, Beijing 100028, China

**Keywords:** social capital, waste separation, informal institution, social learning, reputation effect

## Abstract

The increasing amount of waste produced has been a challenge for human health and the environment, causing a call for effective waste management measures in which household waste separation is of great significance. Although an expanding body of literature has examined the impact of social capital on individual waste-separation behavior, few studies have explicitly discussed the endogeneity problem and the influence mechanisms. Accordingly, our study investigates the effect of social capital on waste-separation behavior and corresponding mechanisms using a national survey dataset of China. The study also reveals the heterogeneity of the influence of individual characteristics on waste-separation behavior. Our results demonstrate that social capital casts a significant positive impact on waste-separation behavior, providing opportunities for individuals’ social learning and strengthening the reputation effect. The heterogeneous effects of social capital reveal that women, higher-educated individuals, and political party members present better waste-separation behavior. Besides, the impact of social capital varies between urban and rural areas and among different age groups. Our study provides empirical evidence for policy making of household waste-separation management in developing countries from the perspective of informal institutions.

## 1. Introduction

With the rapid global economic and social development in the past few decades, the amount of waste produced has been growing at an increasing rate. The amount of global waste is predicted to grow from about 2.02 billion tons in 2016 to 3.40 billion tons by 2050, and the amount in low- and middle-income countries is expected to increase by about 40% [1]. China became the most significant global waste producer in 2004, and it currently produces 70% of the waste in East Asia and the Pacific Ocean area [2]. The waste produced by 196 large- and medium-sized cities in China reached 0.236 billion tons in 2019 [3]. It also predicts that China will more than double the U.S. solid waste by 2030 [2]. To address the urgent waste-management problem, China and many other counties have adopted waste-management policies and regulations [4,5]. Twelve out of the 17 UN Sustainable Development Goals are directly linked to solid waste management [6]. 

In the process of waste management, waste separation is of great importance. Effective waste separation can significantly improve resource utilization and reduce environmental pollution, contributing to public health, carbon emission reduction, and sustainable development [7,8]. However, there is still a significant gap in waste separation between developed and developing countries [9,10]. For example, without a sound waste-management system available in many developed countries, the process of waste separation and recycling in China remains inefficient: some profitable household waste is sold to scrap dealers, while the rest is often disposed of with landfills or incineration. Therefore, the efficiency of waste-separation facilities in many cities is relatively low [11].

Household behavior is at the crux of the waste-separation process, which is important to the success or failure of waste management as a whole. Studies have disclosed that as the marginal benefit of waste-separation investment presents a downward trend, future investment focus should shift from adding separation facilities to encouraging voluntary household waste separation [12]. Nevertheless, it is challenging to put the rule of waste separation into residents’ practice, for individuals are often inclined to free-rider behavior [13]. Formal law cannot function well alone to promote household waste separation, as informal institutions are necessarily complementary and indispensable to the formal institutions [14,15]. As one of the informal institutions, social capital plays a vital role in waste-separation behavior [16]. Particularly, when implementing waste separation in a relational society such as China [17,18], residents’ social environment and social capital cannot be ignored. Therefore, this paper investigates whether social capital can contribute to forming waste-separation behavior and explores the channels of social capital influencing waste-separation behavior, contributing to finding more effective measures in the waste-separation practice.

Existing literature has identified that social capital can promote public environmental protection and management [19,20]. Furthermore, social capital is significantly associated with waste-separation behavior in urban areas [16,21]. Most of the existing studies apply case study methods or employ regional survey data to analyze the impact of social capital on waste separation in China. For instance, some use the survey data in a couple of provinces or cities in China to examine the waste-treatment method [22,23,24], and some discuss the waste separation and recycling patterns only in a specific city [25]. There are also a few studies using national survey data. However, they have not discussed the endogenous problem of social capital [26,27]. In addition, as far as we know, no previous study has employed an empirical test to reveal the mechanisms of social capital influencing an individual’s waste-separation behavior. Although Li and Wang (2021) theoretically analyzed the mechanisms of how social capital affects the performance of household waste separation in urban communities, the study did not perform any empirical analysis.

Different from the previous literature that uses regional data or case studies, our study, utilizing the data of the Chinese General Social Survey (CGSS), otherwise applied the instrumental variable (IV) technique to dealing with the potential endogeneity problem in assessing the impact of social capital on waste separation and further explored the influence mechanisms. Referring to Shi et al. [28] and Jia and Zhao [24], we measured social capital from three perspectives: social networks, social trust, and social participation. Based on the previous literature, we chose the parental education level as the instrumental variable to discuss and deal with the endogenous problem between social capital and waste separation. Compared with the existing literature, our study further empirically proposes and analyzes two influence mechanisms. The results reveal that social capital encourages individuals’ waste-separation behavior via increasing social learning opportunities and forming external constraints. Furthermore, the study discloses the heterogeneity of social capital effects regarding communities, education levels, age cohorts, gender, and urban–rural areas. Our findings for China are constructive for implementing effective household waste-separation policies and might benefit other developing countries confronting similar problems.

The study contributes to the existing literature in the following three aspects. First, instead of adopting small-scale survey data or case studies, our study adopted national representative data to examine the impact of social capital on waste-separation behavior. Second, we employed the IV technique to correct the endogeneity problem. Third, this paper further analyzes the mechanisms and provides empirical evidence for implementing waste-separation policies.

## 2. Hypotheses

Social capital is a key to solving the dilemma of collective action [29,30]. As an informal institution, social capital posts a particular impact on individual decision making and behavior and plays an essential role in inhibiting air or water pollution [31,32,33]. It can significantly raise people’s awareness of environmental protection, thus encouraging their pro-environment behavior. For example, residents with better community relationships are more environmentally friendly [21]. Several regional studies have found that peasants with more social capital show stronger environmental willingness when dealing with straw waste [23]. Specifically, farmers with a higher frequency of social exchanges, higher institutional trust, or more active participation in rural waste management attain a higher frequency of waste separation [24]. However, it is also found that the impact of individual social capital (including trust, justice, and participation) on environmental governance performance is not significant because an atmosphere of protecting the environment has not been formed in society [34]. Based on the above evidence-adopting case studies or local survey data, we speculate that social capital may generally positively impact individual waste-separation behavior when using national representative survey data. We posit the following hypothesis:

**Hypothesis** **1.** *Social capital can promote individual waste-separation behavior.*

Different dimensions of social capital (social networks, social trust, and social participation) may have various effects on waste separation behavior. Zheng et al. [35] conducted an online survey of 259 subjects and found that social network connections encourage waste-separation behavior. Halkos and Jones’s [36] study also suggested that social trust produces significant positive effects on individual waste-separation behavior in Sweden. Furthermore, Nguyen et al. [37] found that social trust serves as a decisive factor in residents’ willingness to segregate waste in Delhi, India. Thus, we propose the following hypotheses:

**Hypothesis** **1a.** *Social networks can promote individual waste-separation behavior.*

**Hypothesis** **1b.** *Social trust can promote individual waste-separation behavior.*

Social participation can contribute to the emergence of pro-social behavior among community residents [38], and those who spend more time on volunteer activities are more likely to spend time on waste management [39]. Thus, we propose the following hypothesis:

**Hypothesis** **1c.** *Social participation can promote individual waste-separation behavior.*

Social capital can decrease the cost of cooperation [20]. The quality and quantity of social capital determine the state of trust and interaction among group members [40]. Specifically, social networks and social participation can provide the opportunity for cooperation, and social trust plays a crucial role in promoting cooperation [41,42]. Furthermore, cooperation can promote social learning, including gaining knowledge from peers and learning during the cooperation process [43]. Therefore, social capital may facilitate learning waste separation knowledge and behavior by promoting cooperation. It is also argued that the lack of knowledge about waste separation become a major obstacle to effective waste separation [44]. By participating in public education activities, individuals can obtain environmental protection knowledge and form norms that affect personal behavior [45]. For example, a study of plastic bag recycling behavior among Ecuadorian residents found that participation in relevant social learning can effectively improve the utilization rate of plastic bags [46]. Thus, we propose the following hypothesis:

**Hypothesis** **2.** *Social capital can promote individual waste-separation behavior by providing social learning opportunities.*

Social capital produces reputation effect, which provides instrumental benefits (including obtaining valuable resources and the potential to influence others) and symbolic benefits (e. g., meeting basic respect needs) [36]. Driving by these gains, individuals may develop more positive waste-separation behavior. Chen and Gao [47] found that mental satisfaction, a kind of symbolic benefit, is one of the essential drivers to motivate residents to segregate waste effectively. At the same time, individuals tend to avoid reputation damage from negative waste-separation behavior. Existing literature has shown that peasants’ reputation demands significantly promote their pro-environmental behavior [48]. We speculate that individuals with more social capital would care more about reputation, thereby showing better waste-separation behavior. We propose the following hypothesis:

**Hypothesis** **3.** *Social capital can influence individuals’ waste-separation behavior through the reputation effect.*

**Figure 1:**
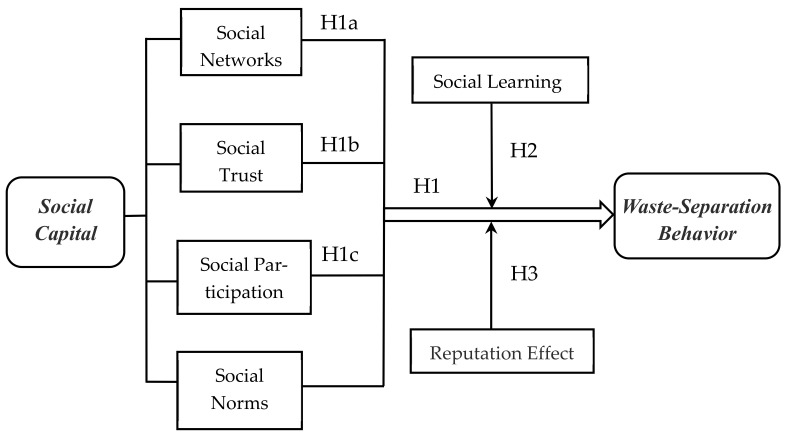
The analytical framework of the social capital impact on waste-separation behavior.

Based on the literature review, we mainly analyzed social capital in three dimensions: social networks, social trust, and social participation. As shown in Figure 1, we propose a research framework based on the above hypotheses to explain the impacts of social capital and its various dimensions on waste separation and the influence mechanisms.

## 3. Data and Empirical Strategy

### 3.1. Data

We utilized the data from the Chinese General Social Survey (CGSS) 2013 conducted by Renmin University of China to explore the effects of social capital on the waste-separation behavior of residents. The sample size is about 12,000 from 134 municipal districts and counties. The questionnaire includes variables at the levels of community, family, and especially the individual in detail. Finally, we retained a sample of 11,282 individuals after cleaning the data.

To reflect individual social networks, we drew on three questions from the CGSS 2013 questionnaire: (1) “How often did you get together with relatives who do not live with you over the last year?”; (2) “How often have you met with your friends over the past year?”; and (3) “The frequency of having entertainment activities with other friends.” We employed the factor analysis method to construct the component index of social networks and extract a factor whose KMO (Kaiser–Meyer–Olkin) test result is 0.60, indicating the factor analysis can be performed. To measure social trust, we selected two questions: “Do you agree that the majority of people in the society can be trusted?” and “Do you trust the strangers in the society?” Adding together these two questions, we generated the social trust variable. Social participation is a dummy variable equal to one if the individual participated in the last neighborhood/village committee elections or has been a labor union member; otherwise, the variable was equal to zero. Finally, using factor analysis again and all the original indicators in the questionnaire, we extracted three common factors, generating the social capital variable. Table 1 provides further details relating to the factor analysis. We show the exploratory factor analysis process of social capital in Appendix A.

Previous studies have verified the impact of demographic characteristics, such as gender, income, and education level, on individual environmental behavior and household waste-management behavior [49,50,51]. This study controlled respondents’ age, gender, marital status, education level, and per capita annual family income. In addition, members of the Communist Party of China (CPC) and eight democratic parties have a stronger sense of social responsibility and more advanced ideological awareness. They are likely to exhibit more pro-environmental behavior, so we controlled for the political status of individuals. Considering that the community environment of respondents varies, and the household waste-management facilities and rules also affect the individual waste-separation behavior, this paper controls the type of community where individuals live. According to the completeness of waste separation facilities, the communities are divided into three categories: poor, general, and good. Specifically, communities with poor waste-sorting facilities refer to rural communities. Communities with average waste sorting facilities include old city communities and new urban communities transformed from rural communities. Furthermore, communities with good waste-sorting facilities include apartments, indemnificatory housings, common commercial housings, senior villa areas, and top-grade residential quarters. The descriptive statistics of the main variables are illustrated in Table 2. In terms of individual characteristics, about 50% of respondents are males, and 79% are married. The samples we use are consistent with the data of the China Statistical Yearbook 2013.

### 3.2. Empirical Strategy

As individual waste-separation behavior is measured using ordered discrete variables, we used the ordered probit model as the benchmark in the following form:

(1) $$y_{i}^{*}=\alpha +\beta SC_{i}+\theta^{\prime }X_{i}+\underset{P}{\sum }\delta_{P}+\varepsilon_{i}\;\;$$

(2) $$y_{i}=\left\{\begin{array}{c} 0,\;\;y_{i}^{*}\leq \mu_{1}\\ 1,\;\;\mu_{1}<y_{i}^{*}\leq \\ 2,\;\;y_{i}^{*}>\mu_{2} \end{array}\right.\mu_{2}$$

$y_{i}{}^{*}$ represents the latent variable, which represents the frequency of individuals’ waste-separation behavior. $SC_{i}$ is the set of variables representing the level of social capital, including social network, social trust, and social participation. $y_{i}$ is the dependent variable measuring the frequency of waste-separation behavior of individual i in the past year. In Equation (2), $\mu_{1}$ and $\mu_{2}$ are two boundary points classifying the household waste separation level into three categories. $y_{i}{}^{*}\leq \mu_{1}(y_{i}=0)$ indicates that individual i never segregates waste; $y_{i}=1$ means that individual i shows separating-waste behavior occasionally; and $y_{i}=2$ means that individual i often segregates waste. $X_{i}$ is the characteristic vector at the individual and household levels. Given that waste facilities and management levels vary in different regions, which can influence individual waste-separation behavior, we controlled for community types of respondents $X_{i}$. We also added the provincial fixed effects $\sum_{p}\delta_{p}$ in the regression to control for the disparity among regions. $\varepsilon_{i}$ is a random disturbance term.

## 4. Results

Table 3 shows the baseline regression results of model 1. Individuals’ social capital positively impacts the frequency of waste separation, which is significant at the 1% level (column 4 of Table 3). Columns 1–3 of Table 3 show that an individual’s social network, social trust, and social participation substantially affect waste-separation behavior. The possible reason is that individuals with higher social capital interact more often with their relatives, friends, or neighbors and are thus more familiar with their living environment and have a stronger sense of social identity. Thereby, individuals may pay more attention to environmental conditions and have a more positive attitude toward environmental protection; therefore, they are more willing to segregate waste. The results provide empirical evidence to support Hypothesis 1.

We further report the marginal effects of each explanatory variable on waste-separation behavior in Table 4. If we take the mean of all variables, for each unit increment in individual social capital, the probability of “never segregating waste” would decrease by 0.036, the probability of “occasionally segregating waste” would increase by 0.020, and the likelihood of “often segregating waste” would increase by 0.016. Among the three dimensions of social capital, the marginal effect of social networks is the most significant. For each unit increment in an individual’s social network, the probability of “never segregating waste” decreases by 0.024, and the probabilities of occasionally and often segregating waste both increase by 0.012, with all of them being significant at the 1% level. Compared to those without social participation, residents involved in public activities are 0.023 less likely never to segregate waste and 0.011 more likely to occasionally or often segregate waste. It can be seen from the regression results in Table 3 and Table 4 that social trust would also promote individual waste separation, but the effect is smaller than social networks and social participation. Controlling for all other variables, for each unit increment in social trust, the probability of “never segregating waste” decreases by 0.007, and the probability of occasionally or often segregating waste increases by 0.003.

The regression results of individual characteristics on waste separation in Table 4 are consistent with the literature. Men are less likely to segregate waste [52]. Men’s probability of “never segregating waste” is 0.031 higher than women’s, and men are 0.014 less likely to segregate waste often. Besides, it is generally believed that more educated individuals are more sensitive to social welfare and would exhibit more pro-environmental behaviors [46,53,54]. As seen in Table 4, compared to illiterate respondents, the probability of “often segregating waste” is on average 0.016 times higher for those who have attended primary school, 0.039 times higher for the group who have attended junior high school, 0.065 times higher for those who have attended high school, and 0.071 times higher for those with a college degree or above.

Having a political identity, such as being a member of the Chinese Communist Party or eight democratic parties, would increase the probability of “often segregating waste” by 0.022, which is significant at the 1% level and consistent with related studies based on the values-beliefs-norms (VBN) model [55,56]. The literature on pro-environmental behavior suggests that the sense of morality and obligation have a significant impact on individual behavior [57,58]. According to universal values, Schwartz’s model of human values divides people into four groups [59,60], among which the altruistic group would engage in pro-environmental behavior more actively [61,62,63]. In addition, the community environment of individuals is associated with waste-separation habits. Residents of communities with average waste-separation facilities have a 0.028 higher probability of “often segregating waste” than rural residents. Residents living in communities with sound waste-sorting facilities will have a 0.061 higher probability of “often segregating waste” than rural residents. The reason may be that people’s waste-separation behavior is related to local environmental institutions and the prevalence of waste-separation facilities. 

We found that social capital can significantly promote individual waste-separation behavior. Nevertheless, there may be endogeneity problems as social capital is influenced by some unobservable omitted variables, such as customs and culture [64]. Since the waste classification is a discrete variable and the two-stage least squares method based on continuous variables may fail, we adopted the Conditional Mixed Process (CMP) proposed by Roodman [65] for regression analysis to deal with the endogenous problem. CMP is a two-stage regression, where the first stage finds the instrumental variables of the core explanatory variables and evaluates their interdependency. The second stage performs regression analysis with the instrumental variables in the model, using the joint likelihood estimation method. Education is the primary channel for intergenerational transmission of socioeconomic status, and parents’ educational experience has a significant positive impact on their offspring’s human capital and socioeconomic status [66]. Therefore, we used “parent_edu” as the instrumental variable for social participation.

We tested the validity of using “parent_edu” as an instrumental variable. First, the correlation between “social participation” and “parent_edu” was tested (Table 5, column 4), and the regression coefficient of “parent_edu” is significant at the 1% level. It means that individuals with higher “parent_edu” are more likely to participate in social activities. In addition, the F-test value of the first-stage regression is 10.9, passing the weak instrument test.

Columns 2–3 of Table 5 report the CMP estimation results and find that social participation still has a positive effect on individual waste-separation behavior after controlling for the endogeneity. We further estimated Equation (1) using the OLS model, as shown in column 1 of Table 5. Moreover, columns 4–5 of Table 5 show the results of the first and second stages of the two-stage least squares (2SLS) estimation. The results of the instrumental variable regression indicate that social participation significantly increases the probability of an individual segregating waste, which is consistent with the conclusion of our OLS regression, suggesting that the estimated coefficients of the OLS regression are underestimated.

## 5. Discussion

### 5.1. Mechanism Tests

The above results have suggested that social capital casts a significant positive impact on individual waste-separation behavior, and we will further discuss the influence mechanisms. First, social capital can increase cooperation opportunities with which one’s environmental protection willingness may be enhanced by social learning [67,68]. Second, social capital may promote environmental behavior by reputation effect [48]. Thus, we speculate that social capital will affect the environmental behavior of residents through the two mechanisms and examine Hypotheses 2 and 3.

#### 5.1.1. Social Learning

We measured social learning by individual participation in various environmental protection activities. Since the product-term test of the mediating effect coefficient method proposed by Sobel [69] is only applicable to the linear mediating effect model, while this paper is a non-linear probability model, we used the OLS method to test the mediating effects after standardizing the dependent variables. Column 1 of Table 6 shows the baseline regression results; columns 2, 4, and 6 show the results of the regression of social capital on social learning; and columns 3, 5, and 7 show the results of the regression after adding the mediator variables. The results reveal that social capital significantly increases the frequency at which individuals discuss environmental issues with their relatives and friends and motivates people to participate more actively in environmental activities organized by the government, affiliate, or private environmental groups, which significantly increases their social learning opportunities. After adding a mediator variable in columns 3, 5, and 7, respectively, the effect of social capital on waste separation is still significantly positive, but the degree of the effect decreases. The mediating effect of social learning is 0.63 (0.143 × 0.293/0.066 = 0.63) when measured by “discussing environmental issues with friends and relatives”, 0.26 when measured by “participating in government environmental activities”, 0.63 when measured by “discussing environmental issues with friends and family”, 0.26 when using “participating in government environmental activities”, and 0.22 when using “participating in private environmental activities”, indicating that by providing social learning opportunities, social capital can increase the frequency of waste separation.

We further tested the social learning mechanism using individuals’ environmental knowledge scores. Individuals’ group learning promotes information exchanges, and the more social learning opportunities individuals have, the more knowledge they would acquire [70]. Therefore, if social capital promotes waste-separation behavior by facilitating social learning, individuals will gain more environmental protection knowledge. From Table 7, it can be seen that there is a significant positive effect of social capital on individuals’ environmental protection knowledge level, and the mediating effect is about 0.18, indicating that social capital does improve individuals’ relevant environmental protection knowledge by increasing the opportunities of social learning to improve the frequency of individual waste-separation behavior. The results support Hypothesis 2.

#### 5.1.2. Reputation Effect

In society, individuals are subject to the external constraints of informal institutions such as reputation, which may lead to more pro-environmental behavior. A study found that peasants’ reputation demand significantly affects their pro-environmental behavior [48]. Furthermore, we deduced that individuals with more social capital care more about personal reputation and thus present better waste-separation behavior. The results in column 2 of Table 8 show that individuals with more social capital more greatly value reputation. Furthermore, the coefficients of “social capital” and “reputation” in column 3 of Table 8 are significantly positive, and the magnitude of the mediating effect is about 0.018, indicating that social capital promotes waste-separation behavior by reputation effect. The results support Hypothesis 3.

### 5.2. Heterogeneous Effects

According to the baseline regression results in Table 3, the coefficient of age on waste-separation behavior is negative and significant, indicating that older groups show less waste-separation behavior than younger groups. To further examine the cohort effect, the sample was divided into five age cohorts: 30 and below, 30–40, 40–50, 50–60, and over 60 years old. As can be seen in panel A of Table 9, the influence of the social network on waste-separation behavior gradually decreases with age. This effect may be because the older cohorts have relatively lower environmental awareness and knowledge; also, they focus more on themselves and their families, which can lead to lower social frequency. The results of panel B and panel C in Table 9 show that the influence of social trust on waste-separation behavior is mainly concentrated in the 50–60-year-old cohort, while the influence of social participation is primarily detected in the 30–40-year-old cohort. In general, the impact of social capital on waste-separation behavior is in cohorts over 30–40 years old, and the impact decreases with increasing age until 60 years old.

As there are significant differences in the social environment, lifestyle, and behavior habits of residents between urban and rural areas in China, the impact of social capital on waste-separation behavior of urban and rural residents may also be different. In the CGSS survey data, respondents’ residences consist of central urban areas, non-central urban areas, urban fringe, towns, and rural areas. This study classified all the respondents living in rural areas to rural residence and the rest to urban residence. According to the results in Table 10, the influence of social networks on waste-separation behavior in rural areas is 0.088, which is larger than 0.059 in urban areas, although both are significant. Social trust exposes a significant impact on the waste-separation behavior of rural residents, whereas social participation otherwise influences urban residents more.

## 6. Conclusions

Based on the national representative survey data CGSS in China, our study demonstrates that social capital is a critical factor affecting individual waste-separation behavior. Specifically, social networks, social trust, and social participation promote individual waste-separation action. To deal with the endogenous problem, we utilized the instrumental variable and found that social participation exerts a causal effect on waste-separation behavior. Furthermore, our examination of influence mechanisms evidences that social capital can improve environmental protection knowledge by providing opportunities for individuals’ social learning and strengthening the reputation effect to encourage residents’ waste-separation behavior. Heterogeneous effects of social capital exist among individuals with different characteristics of gender, age, education level, political identity, and living area.

Our results provide valuable policy implications on improving waste-separation management, especially for developing countries plagued by environmental pollution. In addition to the formal institution construction and technological innovation, the government should also give full play to informal institutions, such as social capital, in promoting residents’ waste-separation behavior by enhancing the interaction, communication, and general trust among residents. First, the government can organize various publicity activities and improve the individual waste-separation knowledge. Second, the government can establish a people supervision system to integrate the concept of environmental protection into social values, thus improving residents’ sense of responsibility for environmental protection and stimulating their motivation to participate in waste separation. Besides, the relevant administration can pay more attention to young and highly educated individuals and give full play to the role model of CPC members and other cadres. At the same time, urban–rural differences should be considered. We should pay attention to improving the social trust of residents in rural areas, making them believe that others also strictly carry out waste separation and be confident to participate in collective activities. For urban residents, more attention should be paid to improving social participation, enhancing ownership, and internalizing environmental protection awareness.

There are some limitations to our study. The national representative data (CGSS2013) needs to be improved to provide more adequate information on waste-separation attitudes and intentions. First, the specific municipal district and county information is not disclosed. Second, this survey lacks variables that can be used to measure social norms accurately. In addition, analysis of the influence mechanisms of social capital is not sufficient. Social capital can lower the threshold of social information interaction and has a significant positive impact on information exchange [71]. Furthermore, social capital can improve the dissemination of waste-separation information, thus increasing the frequency of waste-separation behavior among urban residents [47]. Nevertheless, we have not empirically tested the information mechanism for the lack of appropriate measurement variables. Further studies can be conducted after obtaining the improved data.

## Figures and Tables

**Table 1:** The results of factor analysis.

Panel A: Social Capital						
Variable	Original Question in the Questionnaire	Factor Loading			KMO	Bartlett Test
		Factor 1	Factor 2	Factor 3		
Together_relative	How often did you get together with relatives who don’t live with you over the last year?	**0.72**	−0.02	0.10	0.60	7637.4 ***
Together_friend	How often have you met with your friends over the past year?	**0.86**	0.02	−0.01		
Activity_friend	The frequency of having entertainment activities with other friends?	**0.73**	0.08	−0.06		
Trust_society	Do you agree that the majority of people in the society can be trusted?	−0.03	**0.82**	0.11		
Trust_stranger	Do you trust the strangers in the society?	0.09	**0.80**	−0.10		
Vote	Have you voted in the last neighborhood committee/village committee election?	−0.13	0.04	**0.70**		
union	Are you a labor unionist?	0.12	−0.02	**0.73**		
**Panel B: Social networks**						
Variable	Original question in the questionnaire		Factor loading	KMO	Bartlett test	
			Factor 1			
Together_relative	How often did you get together with relatives who don’t live with you over the last year?		0.72	0.60	6023.8 ***	
Together_friend	How often you have met with your friends over the past year?		0.86			
Activity_friend	The frequency of having entertainment activities with other friends?		0.75			

Note: The data in Table 1 are all quoted from the CGSS2013; the data were positively processed; ***, **, * denotes significant levels at 1%, 5%, and 10%, respectively.

**Table 2:** Descriptive statistics of variables in the model.

Variable	Variable Descriptions	Mean	Std. Dev.	Min	Max
***Social networks***					
Together_relative	How often did you get together with relatives who do not live with you over the last year?	2.25	0.72	1	5
Together_friend	How often have you met with your friends over the past year?	2.41	0.93	1	5
Activity_friend	The frequency of having entertainment activities with other friends?	4.13	1.78	1	7
Networks index	Social networks index = factor loading × variance contribution ratio	3.73	1.16	1.28	7.22
***Social trust***					
Trust_society	Do you agree that the vast majority of people in the society can be trusted?	3.28	1.03	1	5
Trust_stranger	Do you trust the strangers in society?	2.59	0.90	1	5
Trust index	An ordered variable summed by subindexes from 0 to 8	3.88	1.57	0	8
***Social participation***					
Vote	Have you voted in the last neighborhood/village committee elections?	0.44	-	0	1
Union	Are you a labor union member?	0.19	-	0	1
Participation index	Social participation index	0.54	-	0	1
Social capital index	Social capital index = factor loading × variance contribution ratio	2.73	0.84	0.82	5.54
Separation	The frequency of waste-separation behavior in the past year (Never = 0, sometimes = 1, often = 2)	0.57	0.70	0	2
Age	age	48.56	16.37	17	97
Male	Male = 1, female = 0	0.50	-	0	1
Married	Married with spouse = 1; other marital status = 0	0.79	-	0	1
***Education level***					
Illiterate	No education = 1, otherwise = 0	0.14	-	0	1
Primary education	Primary school = 1, otherwise = 0	0.21	-	0	1
Junior high school	Junior high school = 1, otherwise = 0	0.29	-	0	1
High school	High school = 1, otherwise = 0	0.19	-	0	1
College and above	College and above = 1, otherwise = 0	0.16	-	0	1
Party membership	Member of Chinese Communist Party or eight democratic parties in China = 1, otherwise = 0	0.10	-	0	1
Lnincome ^(1)^	Yuan, log of annual household income per capita	10.29	2.31	0	16.12
***Type of community***					
Community_poor	Rural communities.	0.41	-	0	1
Community_average	Old city communities and new urban communities transformed from rural communities.	0.25	-	0	1
Community_good	Apartments, indemnificatory housings, common commercial housings, senior villa areas, and top-grade residential quarters.	0.33	-	0	1
Parent_edu	The highest educational level of parents (years).	4.95	4.85	0	19
***Social learning***					
Discussion	The frequency of discussing environmental issues with relatives and friends?	0.56	0.63	0	2
Act_gov	The frequency of participating in environmental activities organized by the government and affiliations?	0.27	0.53	0	2
Act_group	The frequency of participating in environmental activities organized by private environmental groups?	0.19	0.45	0	2
Knowledge	The correct number of ten environmental protection knowledge judgment questions.	3.09	1.96	0	10
Reputation	Do you think it is important for you to be respected in society? (The demand degree ranging from low to high is divided into 8 options)	2.46	0.90	1	8

Notes: ^(1)^ Annual household income per capita is calculated by dividing total household income in a year by the number of people in the household who live with the respondent and are not economically independent of each other, and 57 samples have zero annual household income per capita; the variables in Table 2 are all quoted from the data of CGSS 2013. The mean and standard deviation of each variable were generated after summing up the 11,282 validated samples.

**Table 3:** Ordered probit model results of social capital on waste-separation behavior.

	(1)	(2)	(3)	(4)
Networks index	0.070 ***			
	(0.01)			
Trust index		0.020 **		
		(0.01)		
Participation index			0.065 *	
			(0.03)	
Social capital index				0.174 ***
				(0.03)
Age	−0.007 ***	−0.008 ***	−0.008 ***	−0.012 ***
	(0.00)	(0.00)	(0.00)	(0.00)
Male	−0.091 ***	−0.088 ***	−0.088 ***	−0.150 ***
	(0.02)	(0.02)	(0.02)	(0.04)
Married	0.015	0.013	0.005	0.020
	(0.03)	(0.03)	(0.03)	(0.05)
Primary education	0.124 **	0.129 ***	0.117 **	0.174 **
	(0.05)	(0.05)	(0.05)	(0.08)
Junior high school	0.264 ***	0.276 ***	0.259 ***	0.411 ***
	(0.06)	(0.06)	(0.06)	(0.10)
High school	0.424 ***	0.445 ***	0.428 ***	0.683 ***
	(0.06)	(0.06)	(0.06)	(0.10)
College and above	0.459 ***	0.482 ***	0.470 ***	0.747 ***
	(0.07)	(0.07)	(0.07)	(0.12)
Party membership	0.146 ***	0.150 ***	0.142 ***	0.236 ***
	(0.04)	(0.04)	(0.04)	(0.07)
Lnincome	0.003	0.004	0.005	0.009
	(0.01)	(0.01)	(0.01)	(0.01)
Community_average	0.178 ***	0.183 ***	0.186 ***	0.296 ***
	(0.06)	(0.06)	(0.06)	(0.10)
Community_good	0.386 ***	0.400 ***	0.405 ***	0.643 ***
	(0.06)	(0.06)	(0.06)	(0.10)
Province Dummy	YES	YES	YES	YES
N	11,282	11,282	11,282	11,282
Pseudo R^2^	0.100	0.099	0.099	0.104

Note: The values in parentheses indicate the standard error of county/street level clustering. Clustered standard errors in parentheses. *** *p* < 0.01, ** *p* < 0.05, * *p* < 0.1.

**Table 4:** Ordered probit model results of marginal effects of social capital on waste separation behavior.

	(1)	(2)	(3)
	Never	Occasionally	Often
Networks index	−0.024 ***	0.012 ***	0.012 ***
	(0.01)	(0.00)	c0)
Trust index	−0.007 **	0.003 **	0.003 **
	(0.00)	(0.00)	(0.00)
Participation index	−0.023 **	0.011 **	0.011 *
	(0.01)	(0.01)	(0.01)
Social capital index	−0.036 ***	0.020 ***	0.016 ***
	(0.01)	(0.00)	(0.00)
Age	0.002 ***	−0.001 ***	−0.001 ***
	(0.00)	(0.00)	(0.00)
Male	0.031 ***	−0.017 ***	−0.014 ***
	(0.01)	(0.00)	(0.00)
Married	−0.004	0.002	0.002
	(0.01)	(0.01)	(0.00)
Primary school	−0.036 **	0.020 **	0.016 **
	(0.02)	(0.01)	(0.01)
Junior high school	−0.085 ***	0.046 ***	0.039 ***
	(0.02)	(0.01)	(0.01)
High school	−0.142 ***	0.077 ***	0.065 ***
	(0.02)	(0.01)	(0.01)
College and above	−0.155 ***	0.084 ***	0.071 ***
	(0.02)	(0.01)	(0.01)
Party membership	−0.049 ***	0.027 ***	0.022 ***
	(0.01)	(0.01)	(0.01)
Lnincome	−0.002	0.001	0.001
	(0.00)	(0.00)	(0.00)
Community_average	−0.061 ***	0.033 ***	0.028 ***
	(0.01)	(0.01)	(0.01)
Community_good	−0.134 ***	0.073 ***	0.061 ***
	(0.02)	(0.01)	(0.01)

Note: The marginal effects reported in Table 4 are based on the regression results in column (4) of Table 3, basically consistent with the marginal effects using social networks, social trust, and social participation as explanatory variables. The marginal effect results based on columns (1), (2) and (3) of Table 3 are presented in Appendix B. The values in parentheses indicate the standard error of town/sub-district level clustering. clustered standard errors in parentheses. *** *p* < 0.01, ** *p* < 0.05, * *p* < 0.1.

**Table 5:** Result of the instrumental variable regression.

	(1)	(2)	(3)	(4)	(5)
	OLS	CMP-probit	CMP-oprobit	2SLS-first	2SLS-second
	Separation	Participation index	Separation	Participation index	Separation
Participation index	0.036 **		0.268 **		2.602 **
	(0.02)		(0.15)		(0.70)
Parent_edu		0.011 **		0.009 ***	
		(0.00)		(0.00)	
Controls	YES	YES	YES	YES	YES
N	11,282	11,282	11,282	10,890	10,890
R^2^	0.177	0.129	0.099	0.159	-
F-statistic	-	-	-	10.902	-

Note: Values in parentheses indicate clustering standard errors. The control variables in the table are consistent with those in the baseline regression in Table 3. Clustered standard errors in parentheses. *** *p* < 0.01, ** *p* < 0.05, * *p* < 0.1.

**Table 6:** Mediating effect of social learning: environmental protection activities.

	(1)	(2)	(3)	(4)	(5)	(6)	(7)
Dependent Variables	Separation	Discussion	Separation	Act_Gov	Separation	Act_Group	Separation
Social capital index	0.066 ***	0.143 ***	0.024 *	0.103 ***	0.049 ***	0.095 ***	0.051 ***
	(0.01)	(0.02)	(0.01)	(0.01)	(0.01)	(0.02)	(0.01)
Discussion			0.293 ***				
			(0.01)				
Act_gov					0.168 ***		
					(0.01)		
Act_group							0.155 ***
							(0.01)
Controls	YES	YES	YES	YES	YES	YES	YES
N	11,282	11,282	11,282	11,282	11,282	11,282	11,282
R^2^	0.176	0.196	0.245	0.163	0.200	0.149	0.196

Note: Clustered standard errors in parentheses. *** *p* < 0.01, ** *p* < 0.05, * *p* < 0.1.

**Table 7:** Mediating effect of social learning: environmental knowledge score.

	(1)	(2)	(3)
	Separation	Knowledge	Separation
Social capital index	0.066 ***	0.241 ***	0.054 ***
	(0.01)	(0.03)	(0.01)
Knowledge			0.050 ***
			(0.01)
Controls	YES	YES	YES
N	11,282	11,282	11,282
R^2^	0.176	0.270	0.183

Note: Clustered standard errors in parentheses. *** *p* < 0.01, ** *p* < 0.05, * *p* < 0.1.

**Table 8:** Mediating effect of reputation.

	(1)	(2)	(3)
	Separation	Reputation	Separation
Social capital index	0.066 ***	0.025 *	0.065 ***
	(0.01)	(0.02)	(0.01)
Reputation			0.050 ***
			(0.01)
Controls	YES	YES	YES
N	11,282	11,282	11,282
R^2^	0.174	0.025	0.178

Note: Clustered standard errors in parentheses. *** *p* < 0.01, ** *p* < 0.05, * *p* < 0.1.

**Table 9:** The heterogeneous effects of social capital on waste-separation behavior.

	(1)	(2)	(3)	(4)	(5)
	Below 30	(30, 40)	(40, 50)	(50, 60)	Over 60
Networks index	0.035	0.074 **	0.072 ***	0.055 **	0.075 ***
	(0.03)	(0.03)	(0.03)	(0.03)	(0.02)
Trust index	0.006	0.012	0.032 *	0.043 **	0.013
	(0.02)	(0.02)	(0.02)	(0.02)	(0.02)
Participation index	−0.048	0.173 ***	0.084	0.004	0.041
	(0.07)	(0.06)	(0.06)	(0.06)	(0.06)
Social capital index	0.043	0.112 ***	0.101 ***	0.076 **	0.110 ***
	(0.05)	(0.04)	(0.03)	(0.04)	(0.03)
N	1811	2046	2442	2140	2843

Note: The control variables in Table 9 are the same as those in the baseline regression in Table 3. Clustered standard errors in parentheses. *** *p* < 0.01, ** *p* < 0.05, * *p* < 0.1.

**Table 10:** The heterogeneous effects of social capital on waste-separation behavior.

	(1)	(2)	(3)	(4)	(5)	(6)	(7)	(8)
	Urban	Rural	Urban	Rural	Urban	Rural	Urban	Rural
Networks index	0.059 ***	0.088 ***						
	(0.02)	(0.02)						
Trust index			0.015	0.030 **				
			(0.01)	(0.02)				
Participation index					0.093 **	0.013		
					(0.04)	(0.05)		
Social capital index							0.087 ***	0.121 ***
							(0.03)	(0.03)
Controls	YES	YES	YES	YES	YES	YES	YES	YES
N	6709	4573	6709	4573	6709	4573	6709	4573
Pseudo R^2^	0.084	0.053	0.083	0.050	0.083	0.050	0.084	0.053

Note: Robust standard errors in parentheses. *** *p* < 0.01, ** *p* < 0.05, * *p* < 0.1.

## Data Availability

The CGSS 2013 database is available in Renmin University of China: http://cgss.ruc.edu.cn/index.htm (accessed on 30 January 2022).

## Appendix A. Exploratory Factor Analysis on Social Capital

The essence of factor analysis is dimensionality reduction. It attributes multiple variables with complex relationships, such as correlations or dependencies, to several common factors through specific statistical methods. In this study, we adopted exploratory factor analysis to process the following items included in social capital variables.

We selected variables potentially related to social capital from the questionnaire, as shown in Table A1, and conducted a preliminary factor analysis of all the following variables. Using principal component analysis for factor extraction, we extracted all the factors with eigenvalues greater than 1, and the common factor was extracted basing on the correlation matrix. We adopted the KMO test, which compares the simple and partial correlation coefficients between the variables, and the Bartlett test generated from the correlation coefficient matrix.

**Table A1:** Survey Questions about Social Capital.

Variables	Original Question in the Questionnaire	Mean	Std. Dev.	Min	Max
Together_relative	How often did you get together with relatives who do not live with you over the last year?	2.25	0.72	1	5
Together_friend	How often have you met with your friends over the past year?	2.41	0.93	1	5
Activity_neighbor	The frequency of having entertainment activities with your neighbors?	3.70	2.07	1	7
Activity_friend	The frequency of having entertainment activities with other friends?	4.13	1.78	1	7
Contact	The frequency of contact with relatives and friends?	0	1	0	1
Trust_society	Do you agree that the vast majority of people in the society can be trusted?	3.28	1.03	1	5
Trust_stranger	Do you trust the strangers in society?	2.59	0.90	1	5
Vote	Have you voted in the last neighborhood committee/village committee election?	0.44	-	0	1
Union	Are you a labor unionist?	0.19	-	0	1

The result of the KMO test is 0.61, and the result of Bartlett’s Sphericity Test is 11,905.822 (df = 36, *p* < 0.001), indicating that there is a suitable common factor for factor analysis. In this study, a common factor was extracted by principal component analysis, and varimax orthogonal rotation was used to obtain the matrix of factor loads (e.g., Table A2), in which factor 1 contains the most indicators. As “Contact” has low factor loadings and factor score coefficient (e.g., Table A3), we eliminated it before further study.

**Table A2:** Rotated Factor Matrix (1).

Variables	Factor 1	Factor 2	Factor 3	Factor 4
Together_friend	0.814			
Activity_friend	0.712			
Together_relative	0.711			
Contact	0.526			
Trust_society		0.814		
Trust_stranger		0.803		
Vote			0.783	
Activity_neighbor			−0.619	
Union				0.873

**Table A3:** Factor Score Coefficient Matrix (1).

Variables	Factor 1	Factor 2	Factor 3	Factor 4
Trust_stranger	−0.003	0.608	−0.032	−0.129
Union	0.140	0.018	0.700	0.246
Together_friend	0.403	−0.043	0.047	−0.155
Together_relative	0.365	−0.045	0.195	−0.112
Activity_neighbor	0.085	0.009	−0.476	0.363
Trust_society	−0.044	0.616	0.062	0.077
Vote	−0.055	−0.035	0.123	0.781
Contact	0.248	0.062	0.075	0.140
Activity_friend	0.317	−0.031	−0.274	0.067

We conducted the factor analysis again to obtain the rotated factor matrix of Table A4. It can be seen that “Activity_neighbor” appears in two common factors. We regarded it as an invalid variable and eliminated it to explore further.

**Table A4:** Rotated Factor Matrix (2).

Variables	Factor 1	Factor 2	Factor 3	Factor 4
Together_friend	0.843			
Activity_friend	0.742			
Together_relative	0.715			
Trust_society		0.813		
Trust_stranger		0.810		
Vote			0.846	
Activity_neighbor			0.588	−0.506
Union				0.838

Three common factors were finally extracted from seven original indicators, and the social capital index was generated. The result of the KMO test is 0.60, and the result of Bartlett’s Sphericity Test is 11,905.822 (df = 36, *p* < 0.001). It can be seen from Table A5 and Table A6 that “Together_friend”, “Activity_friend”, and “Together_relative” belong to the first public factor, “Social network”; “Trust_ society” and “Trust_stranger” belong to the second public factor, “Social trust”; and “Union” and “Vote” are strong interpretations of the third public factor, “Social participation”.

**Table A5:** Rotated Factor Matrix (3).

Variables	Factor 1	Factor 2	Factor 3
Together_friend	0.860		
Activity_friend	0.734		
Together_relative	0.723		
Trust_society		0.815	
Trust_stranger		0.805	
Union			0.728
Vote			0.699

**Table A6:** Factor Score Coefficient Matrix (2).

Variables	Factor 1	Factor 2	Factor 3
Trust_stranger	0.016	0.609	−0.097
Union	0.072	−0.034	0.691
Activity_friend	0.397	0.028	−0.068
Together_friend	0.468	−0.021	−0.019
Together_relative	0.395	−0.045	0.092
Trust_society	−0.049	0.620	0.097
Vote	−0.064	0.030	0.662

## Appendix B. Results of Marginal Effects Analysis

This section reports more detailed results of the marginal effects analysis. Table A7, Table A8 and Table A9 show the results of the marginal effects of each control variable with the social network, social trust, and social participation as the explanatory variables.

**Table A7:** Marginal Effects of Social Networks on Waste-Separation Behavior.

Variables	(1)	(2)	(3)
	Never	Occasionally	Often
Networks index	−0.024 ***	0.012 ***	0.012 ***
	(0.01)	(0.00)	(0.00)
Age	0.002 ***	−0.001 ***	−0.001 ***
	(0.00)	(0.00)	(0.00)
Male	0.031 ***	−0.016 ***	−0.016 ***
	(0.01)	(0.00)	(0.00)
Marry	−0.005	0.003	0.003
	(0.01)	(0.01)	(0.01)
Primary education	−0.043 **	0.021 **	0.022 **
	(0.02)	(0.01)	(0.01)
Junior high school	−0.091 ***	0.045 ***	0.046 ***
	(0.02)	(0.01)	(0.01)
High school	−0.147 ***	0.073 ***	0.075 ***
	(0.02)	(0.01)	(0.01)
College and above	−0.159 ***	0.078 ***	0.081 ***
	(0.02)	(0.01)	(0.01)
Party membership	−0.051 ***	0.025 ***	0.026 ***
	(0.01)	(0.01)	(0.01)
Lnincome	−0.001	0.001	0.001
	(0.00)	(0.00)	(0.00)
Community_average	−0.062 ***	0.030 ***	0.031 ***
	(0.01)	(0.01)	(0.01)
Community_good	−0.134 ***	0.066 ***	0.068 ***
	(0.02)	(0.01)	(0.01)

Note: The values in parentheses indicate the standard error of county/street level clustering; clustered standard errors in parentheses. *** *p* < 0.01, ** *p* < 0.05, * *p* < 0.1.

**Table A8:** Marginal Effects of Social Trust on Waste-Separation Behavior.

Variables	(1)	(2)	(3)
	Never	Occasionally	Often
Trust index	−0.007 **	0.003 **	0.003 **
	(0.00)	(0.00)	(0.00)
Age	0.003 ***	−0.001 ***	−0.001 ***
	(0.00)	(0.00)	(0.00)
Male	0.031 ***	−0.015 ***	−0.015 ***
	(0.01)	(0.00)	(0.00)
Marry	−0.004	0.002	0.002
	(0.01)	(0.01)	(0.01)
Primary education	−0.045 ***	0.022 ***	0.023 ***
	(0.01)	(0.01)	(0.01)
Junior high school	−0.096 ***	0.047 ***	0.049 ***
	(0.02)	(0.01)	(0.01)
High school	−0.155 ***	0.076 ***	0.078 ***
	(0.02)	(0.01)	(0.01)
College and above	−0.168 ***	0.083 ***	0.085 ***
	(0.02)	(0.01)	(0.01)
Party membership	−0.052 ***	0.026 ***	0.026 ***
	(0.01)	(0.01)	(0.01)
Lnincome	−0.002	0.001	0.001
	(0.00)	(0.00)	(0.00)
Community_average	−0.064 ***	0.031 ***	0.032 ***
	(0.01)	(0.01)	(0.01)
Community_good	−0.139 ***	0.069 ***	0.070 ***
	(0.02)	(0.01)	(0.01)

Note: The values in parentheses indicate the standard error of county/street level clustering; clustered standard errors in parentheses. *** *p* < 0.01, ** *p* < 0.05, * *p* < 0.1.

**Table A9:** Marginal effects of social participation on waste-separation behavior.

Variables	(1)	(2)	(3)
	Never	Occasionally	Often
Participation index	−0.023 **	0.011 **	0.011 *
	(0.01)	(0.01)	(0.01)
Age	0.003 ***	−0.001 ***	−0.001 ***
	(0.00)	(0.00)	(0.00)
Male	0.031 ***	−0.015 ***	−0.016 ***
	(0.01)	(0.00)	(0.00)
Marry	−0.002	0.001	0.001
	(0.01)	(0.01)	(0.01)
Primary education	−0.041 **	0.020 **	0.021 **
	(0.02)	(0.01)	(0.01)
Junior high school	−0.091 ***	0.045 ***	0.046 ***
	(0.02)	(0.01)	(0.01)
High school	−0.150 ***	0.073 ***	0.075 ***
	(0.02)	(0.01)	(0.01)
College and above	−0.164 ***	0.081 ***	0.083 ***
	(0.02)	(0.01)	(0.01)
Party membership	−0.049 ***	0.024 ***	0.025 ***
	(0.01)	(0.01)	(0.01)
Lnincome	−0.002	0.001	0.001
	(0.00)	(0.00)	(0.00)
Community_average	−0.065 ***	0.032 ***	0.033 ***
	(0.01)	(0.01)	(0.01)
Community_good	−0.141 ***	0.070 ***	0.071 ***
	(0.02)	(0.01)	(0.01)

Note: The values in parentheses indicate the standard error of county/street level clustering; clustered standard errors in parentheses. *** *p* < 0.01, ** *p* < 0.05, * *p* < 0.1.
